# ETHNIC DIFFERENCES IN THE RELATIONSHIP BETWEEN SOCIAL CAPITAL AND PSYCHOLOGICAL DISTRESS IN OLDER CALIFORNIANS

**DOI:** 10.1093/geroni/igz038.3092

**Published:** 2019-11-08

**Authors:** Nenette A Caceres, Qais Alemi, Larry Ortiz, Carl Stempel

**Affiliations:** 1 Loma Linda University, Loma Linda, California, United States; 2 California State University, East Bay, Hayward, California, United States

## Abstract

Seniors aged 65 and older are at great risk of psychological distress given their functional decline, which is known to limit participation and engagement in community life. The purpose of this study is to examine whether higher indices of social capital have a positive impact on the mental health of older, ethnic Californians. We conducted a secondary analysis of data for 7,485 Californians 65 and older from the 2016 California Health Interview Survey (CHIS). A principal components analysis generated two social capital measures; one measuring safety and social cohesion, the other civic engagement. Hierarchical linear regression analyses were conducted to assess the independent effects of social capital subscales on the severity of psychological distress as measured by the Kessler-6 (K6).Respondents were on average moderately distressed, with small yet significantly higher K6 scores observed among African Americans, Asians, and Native Americans. The addition of our social capital variables in subsequent steps resulted in little yet significant change in explaining psychological distress (ΔR2 = .02, p < .001) with only neighborhood safety and social cohesion being inversely associated with K6 (β = -.15, p < .001). The interaction between ethnicity and neighborhood safety and social cohesion resulted in non-significant associations with K6 scores for all ethnic minority subgroups; however, for African Americans the relationship with psychological distress actually increased significantly (β = .24, p < .001). Our findings suggest that specific types of social capital may be helpful in remediating psychological distress for certain ethnic minority groups.

